# The many faces of solitary fibrous tumor; diversity of histological features, differential diagnosis and role of molecular studies and surrogate markers in avoiding misdiagnosis and predicting the behavior

**DOI:** 10.1186/s13000-021-01095-2

**Published:** 2021-04-20

**Authors:** Muhammad Usman Tariq, Nasir Ud Din, Jamshid Abdul-Ghafar, Yong-Koo Park

**Affiliations:** 1 Section of Histopathology, Department of Pathology and Laboratory Medicine, Aga Khan University Hospital, Karachi, Pakistan; 2 Department of Pathology and Clinical Laboratory, French Medical Institute for Mothers and Children (FMIC), Kabul, Afghanistan; 3 Emeritus Professor, Kyung Hee University, School of Medicine Vice President of Asia, International Academy of Pathology, U2Labs, Jangwon Medical Foundation 68 Geoma-ro, Songpa-gu, Seoul, 05755 South Korea

**Keywords:** Solitary fibrous tumor, Hemangiopericytoma, STAT-6, NAB2-STAT6, CD34, Staghorn, fusion transcript, Immunohistochemistry

## Abstract

**Background:**

Solitary Fibrous Tumor (SFT) is a distinct soft tissue neoplasm associated with NAB2-STAT6 gene fusion. It can involve a number of anatomic sites and exhibits a wide spectrum of histological features.

**Main body:**

Apart from diversity in morphological features seen even in conventional SFT, two histologic variants (fat-forming and giant cell-rich) are also recognized. In addition, a malignant form and dedifferentiation are well recognized. Owing to diverse histological features and involvement of diverse anatomic locations, SFT can mimic other soft tissue neoplasms of different lineages including schwannoma, spindle cell lipoma, dermatofibrosarcoma protuberans, liposarcoma, gastrointestinal stromal tumor (GIST), malignant peripheral nerve sheath tumor (MPNST), and synovial sarcoma. SFT is classified as an intermediate (rarely metastasizing) tumor according to World Health Organization Classification of Tumors of Soft tissue and Bone, 5th edition. The management and prognosis of SFT differs from its malignant mimics and correct diagnosis is therefore important. Although SFT expresses a distinct immunohistochemical (IHC) profile, the classic histomorphological and IHC profile is not seen in all cases and diagnosis can be challenging. NAB2-STAT6 gene fusion has recently emerged as a sensitive and specific molecular marker and its IHC surrogate marker signal transducer and activator of transcription 6 (STAT6) has also shown significant sensitivity and specificity. However, few recent studies have reported STAT6 expression in other soft tissue neoplasms.

**Conclusion:**

This review will focus on describing the diversity of histological features of SFT, differential diagnoses and discussing the features helpful in distinguishing SFT from its histological mimics.

## Background

The morphological features of solitary fibrous tumor (SFT) were originally described in 1931 by Klemperer and Rabin in a series of 5 cases of pleural neoplasms [1]. Similar tumors were named “solitary (localized) mesothelioma of pleura” by Stout and Murray in 1942 [2]. These tumors were renamed “solitary fibrous tumor” by Stout and Hamidi in 1951 [3]. The term “Hemangiopericytoma” (HPC) was used for the first time by Stout and Murray in 1942 while describing a series of 9 cases [4]. The diagnostic criteria for HPC were refined and features for assessment of malignancy were established by Enzinger and Smith in a large study of 106 cases [5]. Until 1990, SFT was reported only in pleura and lung [6]. The first series of extra-thoracic SFT was published in 1991 [7]. Even before the identification of a unifying molecular signature, owing to their clinicopathological similarities, SFT and HPC were considered to represent two ends of histomorphological spectrum of a single tumor entity [8, 9]. The molecular hallmark of these tumors is the recurrent fusion of NAB2 and STAT6 genes located at chromosomal region 12q13 [10–12]. These tumors were merged together into a single entity in 4th edition of World Health Organization (WHO) Classification of Tumors of Soft Tissue and Bone [13]. Current WHO Classification of Soft Tissue and Bone Tumors has classified SFT as a fibroblastic neoplasm with intermediate (rarely metastasizing) behavior [14]. However, in the current WHO Classification of Tumors of Central Nervous System, extrameningeal SFT and HPC are described as a single group but different histologic grades are assigned to these tumors while retaining the names [15].

## Main text

### Clinical features

SFTs can occur in patients over a wide age range with peak incidence in 5th and 6th decades of life. These tumors are rare in children and adolescents [14, 16]. SFTs have been reported at almost every anatomic site. Pleura is the most common site and accounting for approximately 30% cases. Other frequently involved sites include meninges (27%), abdominal cavity (20%), trunk (10%), extremities (8%) and head & neck (5%) [17]. Pleural SFTs present at somewhat older age (mean = 60.2 years) compared to meningeal (mean = 50.6 years) and extra-pleural SFTs (mean = 50.3 years) [18]. No known risk factors for development of SFTs are currently known [16]. SFTs are usually asymptomatic, slow growing tumors which are often discovered as an incidental finding on imaging [19]. These tumors sometimes produce symptoms related to pressure effects on adjacent tissues/organs [14].

Tumor size can range from 1 to 40 cm with median size of 5–8 cm [14, 19]. SFTs of head and neck are generally smaller in size than the more slowly growing tumors of abdominal cavity may reach larger size over a longer time period before causing significant symptoms [14, 20]. SFTs usually involve deep soft tissues but few tumors may occur in superficial locations [21, 22]. Some SFTs present with “Doege-Potter Syndrome”, a paraneoplastic hypoglycemic syndrome resulting from excessive production of insulin-like growth factor II (IGF2) [23, 24].

### Gross features

**Fig. 1:**
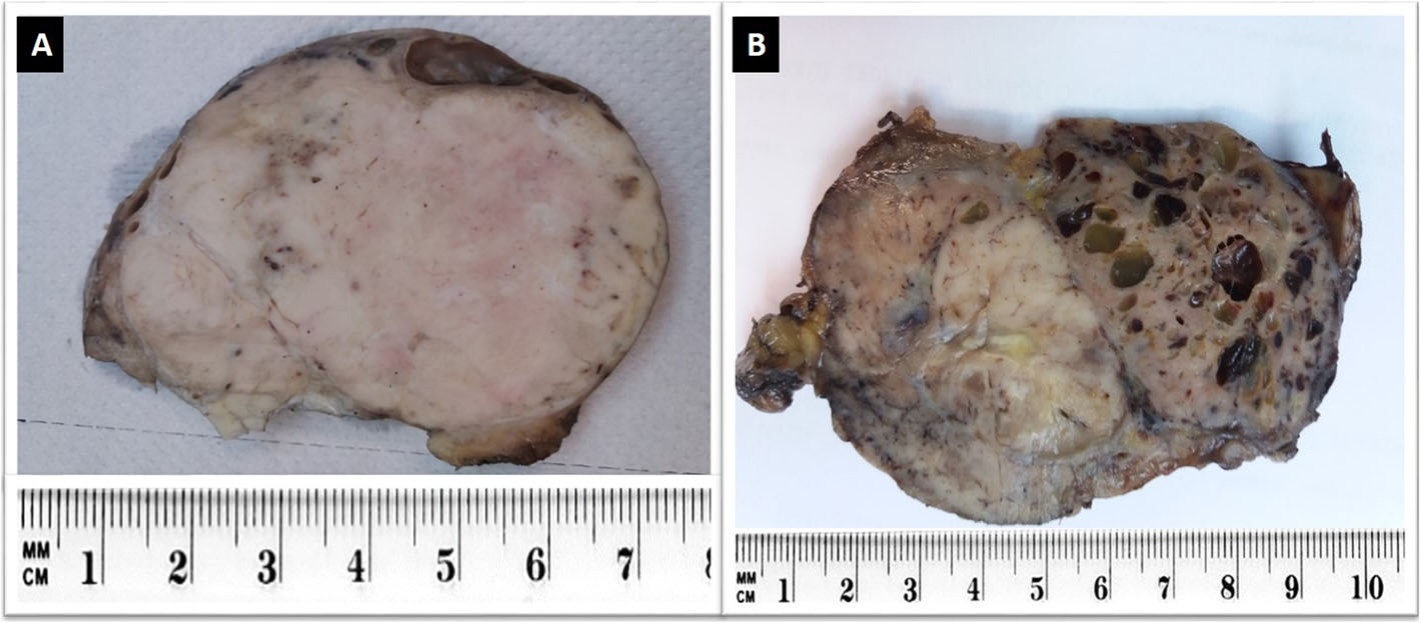
Gross appearance of SFT: **a**. Benign SFT appearing as a well-circumscribed, white and firm tumor. **b**. Malignant SFT of retroperitoneum exhibiting ill-defined borders, variegated cut surface and cystic degeneration

Grossly, conventional SFTs are often well-circumscribed and partially encapsulated with a multinodular, whitish, firm cut surface **(**Fig. 1a). Myxoid change and hemorrhage may be seen in some cases. Malignant and locally aggressive tumors may show irregular infiltrative borders and necrotic areas (Fig. 1b) [14, 17].

### Histological features

**Fig. 2:**
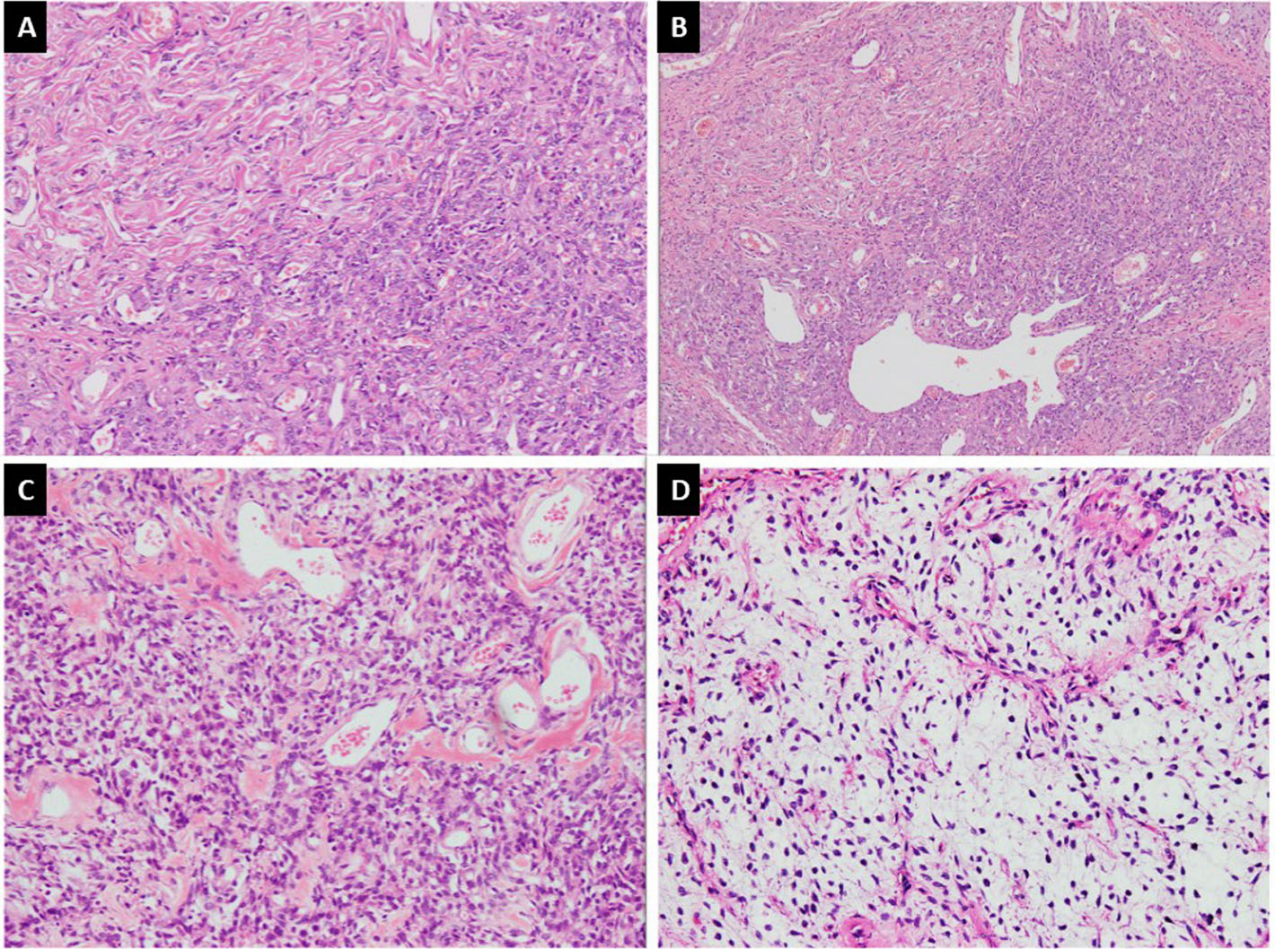
**a**. SFT exhibiting hypo and hypercellular areas of spindle cells against collagenous background stroma along with, **b**. HPC-like vessels, **c**. stromal and perivascular fibrosis and, **d**. myxoid change in stroma

**Fig. 3:**
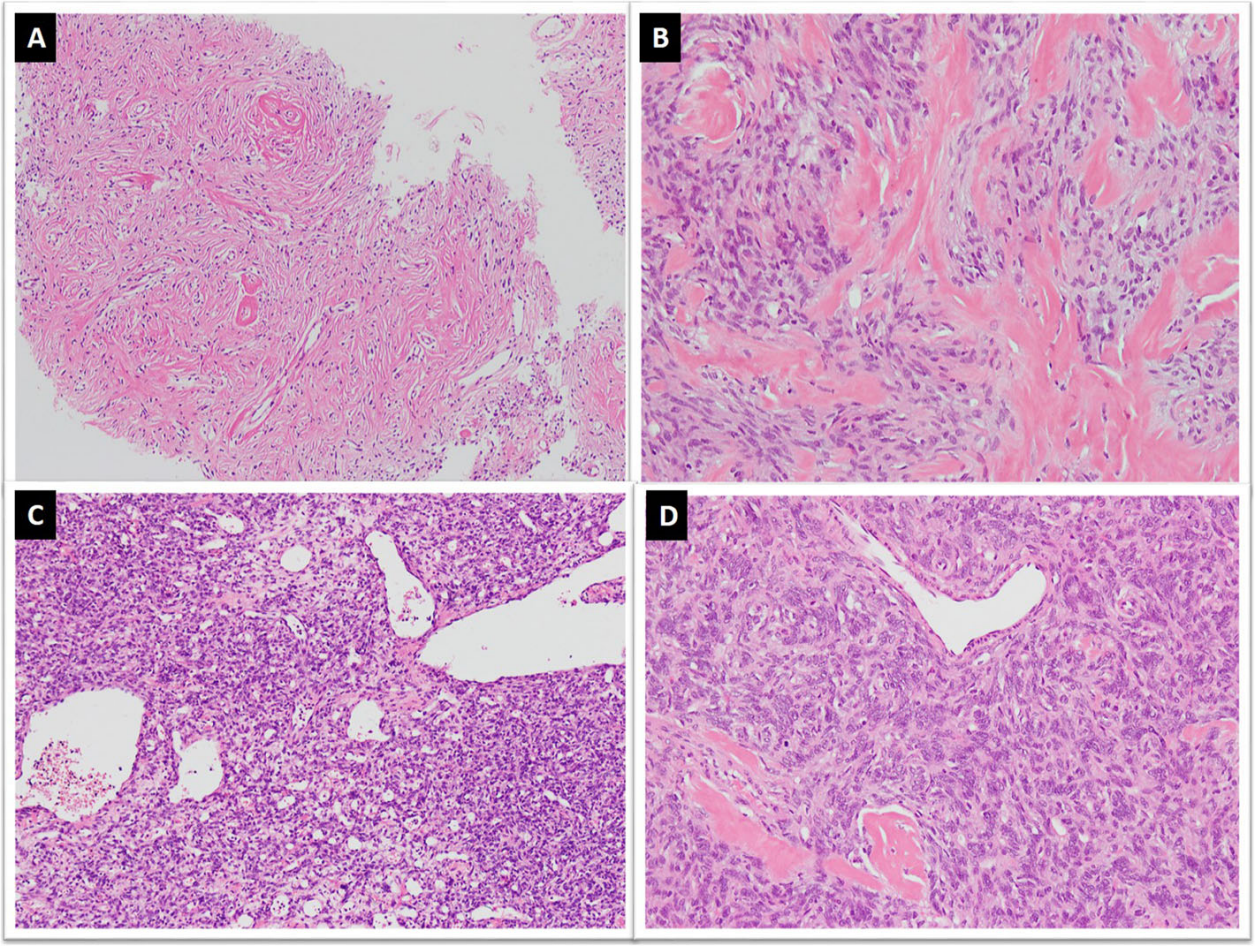
**a**. Classic SFT exhibiting cracking artifact and, **b**. abundant keloid-type collagen, **c**&**d**. Cellular SFT exhibiting increased cellularity, gaping blood vessels and more darkly stained nuclei

**Fig. 4:**
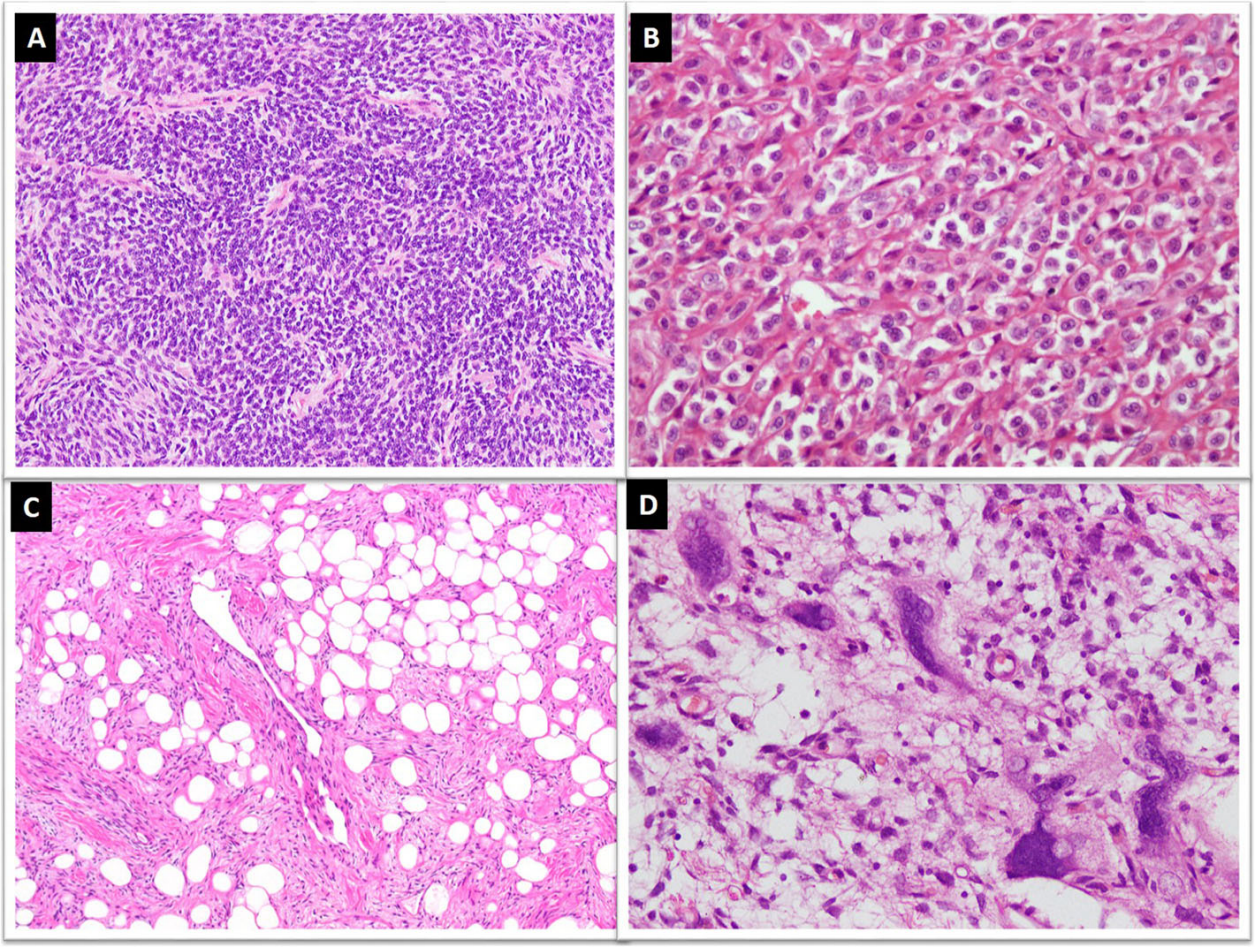
**a**. Markedly cellular tumor showing sheets of small sized cells with hyperchromasia, **b**. Tumor cells exhibiting round cell morphology, **c**. Lipomatous SFT composed of mature adipocyte intermixed with tumor cells, **d**. Giant cell SFT exhibiting multinucleated giant cells focally lining pseudovascular spaces

Histologically, SFTs are variably cellular tumors composed of ovoid to spindled cells exhibiting patternless growth or a storiform pattern against a variably collagenous background stroma containing thin-walled large branching, “staghorn”-shaped (HPC-like) blood vessels **(**Fig. 2a&b). Medium sized blood vessels with perivascular fibrosis are also commonly seen (Fig. 2c) [17, 25]. The background stroma may show focal or diffuse myxoid change (Fig. 2d) [25–27]. Classic fibrous SFTs are paucicellular tumors composed of spindle shaped cells arranged in short wavy fascicles or haphazardly present against prominent fibrous stroma with cracking artifact and abundant keloid-type collagen (Fig. 3a&b). These cells have uniform, elongated or fusiform nuclei and scant cytoplasm. Cellular SFTs are more cellular and composed of spindle shaped and rounded cells with round to oval nuclei having condensed chromatin. Perivascular fibrosis is frequently present. Gaping blood vessels are more frequently seen at the periphery of tumors (Fig. 3c&d). Markedly cellular tumors (still known as HPCs in meninges) are composed of sheets of more primitive-appearing rounded cells (Fig. 4a&b).

The vascular channels in SFT lack a connective tissue layer and the endothelial cells merge with surrounding tumor cells. Fibrotic background and perivascular fibrosis are not seen [20]. HPC-like vasculature can be seen in other mesenchymal tumors including various soft tissue sarcomas [5, 10]. This gave rise to a debate whether HPC was a distinct tumor entity or simply a non-specific histomorphological pattern seen in various tumor types [28–30].

In comparison to pleural and extra-pleural tumors, meningeal tumors are more frequently cellular and show HPC type morphology [18]. SFTs generally show low mitotic activity and lack significant nuclear pleomorphism and/or necrosis [14].

Some variants of SFTs show prominence of certain unusual morphological features. These include fat-forming, giant cell-rich and dedifferentiated variants [17]. The fat-forming variant of SFT or lipomatous HPC shows abundance of mature adipocytes in stroma (Fig. 4c). This variant usually involves deep soft tissues but has been reported in orbit, neck, parotid gland, mediastinum, stomach, retroperitoneum, paratesticular soft tissue and thigh [31–34]. The giant-cell variant of SFT shows many multinucleated stromal giant cells usually arranged around pseudovascular spaces (Fig. 4d). This variant was previously known as giant cell angiofibroma and most of these cases involved periorbital soft tissue. However, it has also been reported in several extra-orbital locations such as head and neck, retroperitoneum, back, vulva, hip, and inguinal region [35–37].

**Fig. 5:**
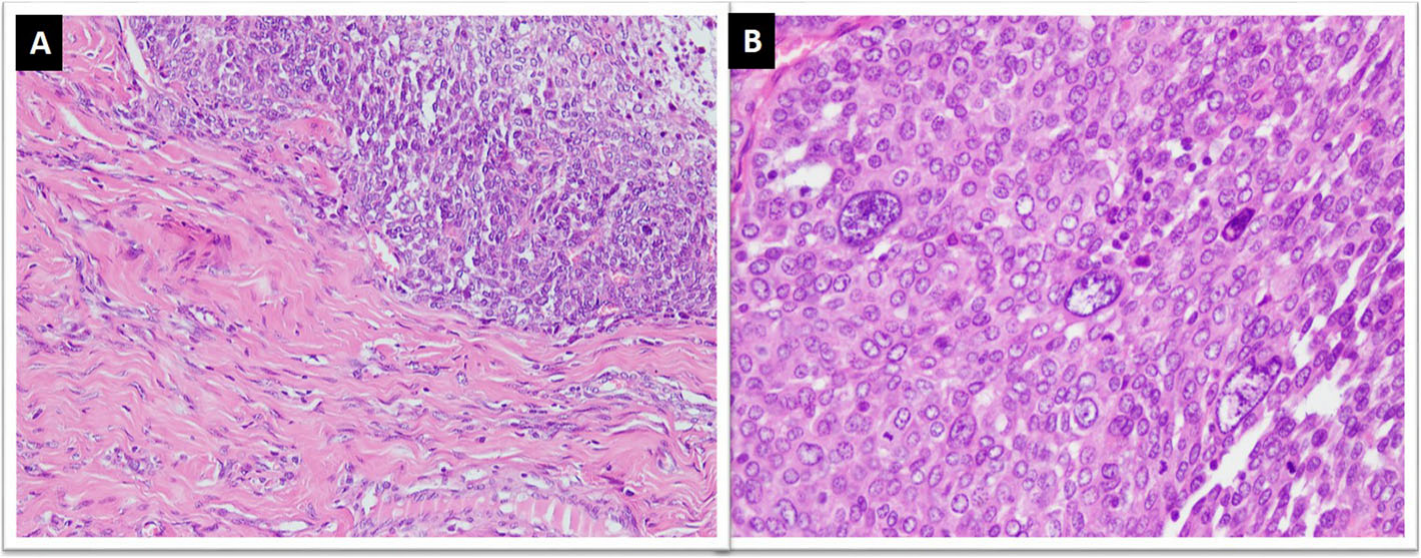
Dedifferentiated SFT; **a**. Abrupt transition of conventional SFT areas with high grade sarcomatous areas with, **b**. marked nuclear pleomorphism and increased mitoses

Like other soft tissue tumors, dedifferentiation is also observed in SFT. The dedifferentiated variant is extremely rare and shows abrupt transition to a low or high-grade sarcoma with adjacent conventional SFT. The dedifferentiated component is mostly in the form of spindle cell sarcoma not otherwise specified or undifferentiated pleomorphic sarcoma (Fig. 5a&b) and can rarely show osteosarcomatous or rhabdomyosarcomatous differentiation [38–43]. Dedifferentiation may be seen in primary or recurrent tumors. It can be associated with loss of expression of immunohistochemical markers and newer molecular alterations. Occasional squamous and neuroendocrine differentiation has also been reported in SFTs [40].

### Features suggestive of malignant behavior

Different clinical and histomorphological features have been described which are suggestive of malignancy. These features include older age, larger tumor size, increased cellularity, increased mitotic activity (≥4/10HPFs or > 2mitoses/2 mm^2^), nuclear pleomorphism, tumor necrosis and infiltrative borders [5, 6, 14, 17, 20, 44–47]. Tumors lacking malignant histological features in primary resection specimens may acquire malignant features at time of recurrence and metastases [47].

In extra-pleural and extra-meningeal tumors, Pasquali et al. found hypercellularity, increased mitotic rate and nuclear pleomorphism to be associated with recurrence and hypercellularity and pleomorphism to be associated with reduced overall survival [48]. Demicco et al. reported patient age, tumor size and mitotic rate to be associated with time to metastasis and tumor related death, and necrosis to be a predictor of metastasis [49]. Tumor size is usually considered to be a negative prognostic factor but SFTs can grow to a large size without behaving aggressively [14, 48]. Kim et al. assessed the utility of different risk assessment systems in SFTs from different sites and found mitotic rate (> 4/10 HPF) to be the only independent prognostic factor [18]. Yamada et al. identified dedifferentiation as a major adverse prognostic factor and hypoglycemia, cerebromeningeal and intra-abdominal locations to be associated with poor prognosis [43].

TP53 immunohistochemical (IHC) expression has also been found to be associated with recurrence and/or metastasis [50]. In one study, TERT promoter mutations were found to be predictors of metastasis-free survival in intermediate risk category of SFT [51]. One of the latest risk stratification models by Demicco et al. is based on assessment of patient’s age, mitoses/mm^2^, tumor size and percentage of tumor necrosis. It stratifies SFTs into low, intermediate and high-risk categories and is more accurate in predicting the prognosis [52].

### Immunohistochemical features of SFT

**Fig. 6:**
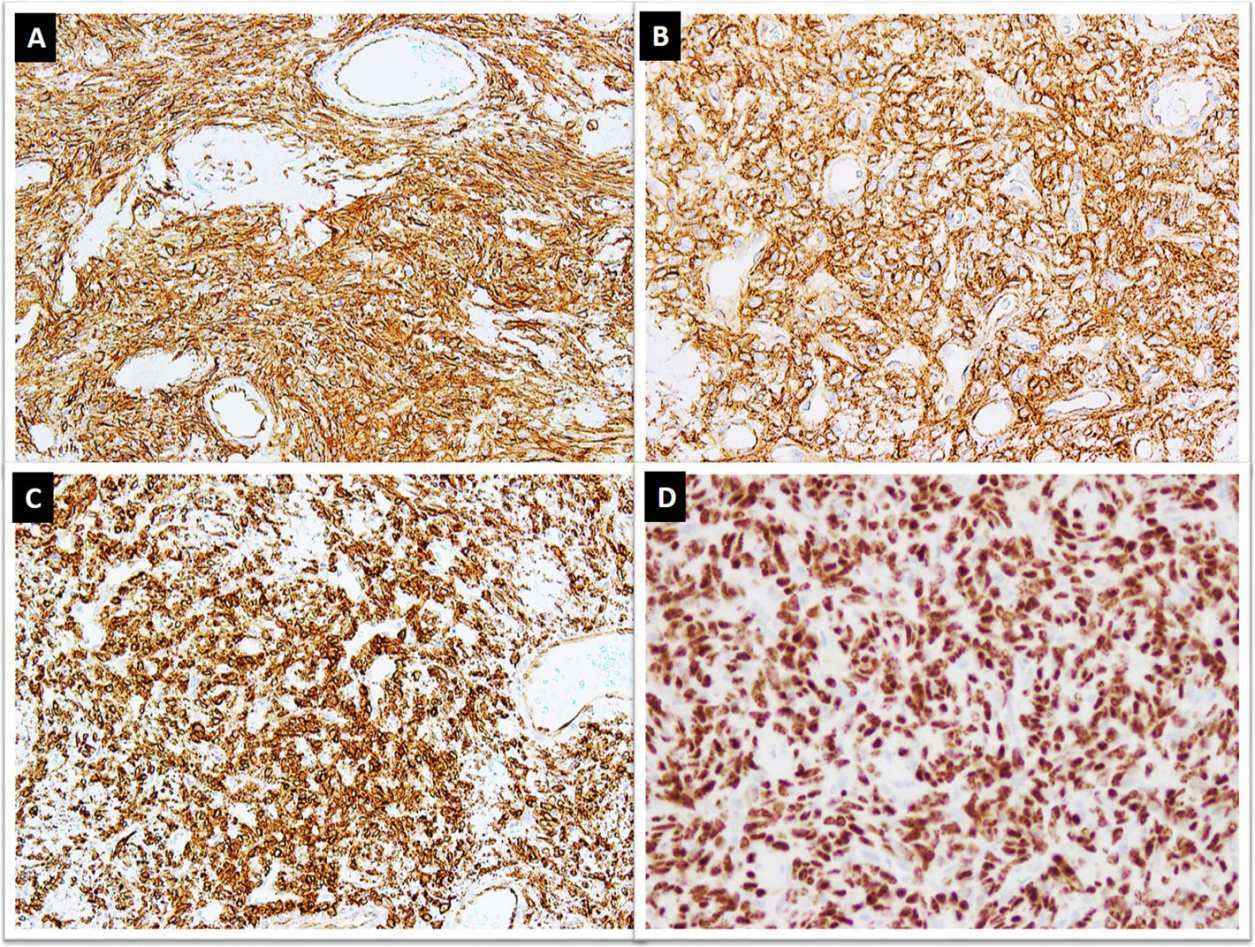
Tumor cells showing positive staining for, **a**. CD34, **b**. CD99, **c**. BCL2 and, **d**. nuclear staining for STAT6 IHC stains

A combination of CD34, CD99 and BCL-2 has been widely used to diagnose SFT. These IHC markers are sensitive and usually show diffuse and strong expression in approximately 90% cases (Fig. 6a-c). However, these markers have limited usefulness due to their expression in other neoplasms closely mimicking SFT histologically [21, 53]. CD34 expression has been observed in 81–95% SFTs but its expression is lost especially in malignant and dedifferentiated tumors [54–57]. BCL-2 is a more sensitive marker (> 90% sensitivity) while CD99 is less sensitive (~ 75% sensitivity). However, specificity of both these markers is quite low [21, 53, 54, 58, 59].

STAT6 IHC stain has emerged as a useful surrogate marker of NAB2-STAT6 gene fusion with excellent sensitivity and specificity and is also expressed in malignant cases (Fig. 6d) [55, 56, 60]. In a recent study, diffuse and strong nuclear expression for STAT6 IHC marker was observed in all 52 cases but gene fusion was detected by reverse transcription polymerase chain reaction in 48 (92%) cases (RT-PCR) [20].

STAT6 IHC marker can also be expressed in some other soft tissue neoplasms such as well differentiated liposarcoma (WDL) or dedifferentiated liposarcoma (DDL), desmoid fibromatosis, unclassifiable sarcoma, neurofibroma, myxoid liposarcoma, undifferentiated pleomorphic sarcoma, low grade fibromyxoid sarcoma, synovial sarcoma (SS) and ovarian fibroma [17, 61, 62]. Doyle et al. observed positive STAT6 expression in 4 out of 35 (11%) cases of DDL [61]. In a large study, Demicco et al. assessed STAT6 expression in 1781 non-SFT mesenchymal tumors and observed strong nuclear expression in 4% cases. Tumors which demonstrated positive expression included unclassifiable sarcoma (8/65{12%}), WDL and DDL (49/409{12%}), desmoid tumor/fibromatosis (14/184{8%}), neurofibroma (3/60 {5%}), clear cell sarcoma (1/19{5%}), myxoid liposarcoma (2/108{2%}) and undifferentiated pleomorphic sarcoma (2/173{1%}). Ouladan et al. evaluated STAT6 expression in 374 non-SFT mesenchymal tumors and positive nuclear staining was observed in only 4 (1%) cases including 2 cases of WDL, 1 case of DDL and 1 case of SS [63]. STAT6 expression was also found in a small subset of non-neoplastic tissue including scar tissue and adipose tissue [62]. In a series of prostatic SFT and smooth muscle tumors of uncertain malignant potential (STUMP), the sensitivity and specificity of STAT6 for SFT was 91 and 75% respectively [64]. The expression of STAT6 in SFT is exclusively nuclear but other tumors may show both nuclear and cytoplasmic staining [62].

Gene expression profiling studies have also identified overexpression of GRIA2 gene and aberrant expression of GRIA2 protein in SFT [12]. This protein is usually expressed in central nervous system tissue and epithelia of some organs but can also be expressed in soft tissue tumors such as SFT and dermatofibrosarcoma protuberans (DFSP) [54]. In one study, overall frequency of GRIA2 IHC expression in SFT was 80%. The frequency was 86% in malignant cases and 100% in dedifferentiated cases [17].

Another study found aldehyde dehydrogenase 1 (ALDH1) to be a useful marker for SFT. It was expressed in 76% cases, and along with STAT6 expression, was found be a useful marker for distinguishing SFT from its soft tissue mimics [63]. ALDH1 was also found to be helpful in differentiating meningeal SFT from meningioma and SS [57].

Some cases of pleural and abdominal SFT may show multifocal expression of cytokeratins [65]. Few cases of SFT may also show focal expression for epithelial membrane antigen (EMA), alpha smooth muscle actin (ASMA), beta catenin, glial fibrillary acidic protein (GFAP) and neuron-specific enolase (NSE). Conversely, expression of h-caldesmon, desmin, CD31 and S100 is almost always negative [65]. TP53 IHC expression is observed in malignant cases [50].

### Differential diagnosis

Owing to diversity of histological patterns exhibited by SFT, Machado et al. termed SFT “the great simulator” of soft tissue tumors [25]. SFTs often pose diagnostic challenge and integration of clinical, histomorphological, immunohistochemical and molecular features is necessary for establishing correct diagnosis [17]. In extra-pleural locations, the pathologist must rule out tumors which are more common in those particular sites [17]. Comparison of clinical, histomorphological, immunohistochemical and molecular features of SFT with some of the tumors important in differential diagnosis is summarized in Table 1.

**Table 1:** Differential Diagnosis of Solitary Fibrous Tumor

	Age (years)	M:F ratio	Sites	Histological features	IHC	Key genetic alterations
**Solitary Fibrous Tumor** [10, 21]	40 to 70	Equal	Pleura Meninges; CNS & spinal cord Deep soft tissues of extremities Abdominal cavity, the pelvis, or the retroperitoneum Head and neck Trunk	Patternless spindled to ovoid cells within a variably collagenous stroma, admixed with branching and hyalinized staghorn-shaped (hemangiopericytomatous) blood vessels.	CD34+, STAT6+	fusion of the *NAB2* and *STAT6* genes
**Synovial Sarcoma, Monophasic** [14, 21]	Adolescents & young adults (2/3rds < 50 years)	1.2:1	Most often deep soft tissues of extremities or limb girdles, distal extremities (fingers, hand foot), Head and & Neck	Fascicles & sheets of uniform spindle cells. May have a herringbone pattern. HPC like branching vessels are common. Often contain stromal hyalinized or wiry collagen bundles	SYT +, TLE1+, EMA+, CK+, STAT6-	SYT-SSX1/2
**Malignant Peripheral** **Nerve Sheath Tumor** [21, 66]	20–50 (median 35)	Equal	Trunk and extremities, followed by the head and neck area	Hypercellular & hypocellular fascicles of spindle-shaped cells (marbleized pattern), perivascular accentuation, HPC-like vascular pattern, increase mitoses, geographic necrosis, heterologous differentiation in 15% of cases	S100 + (< 50%), SOX10 + (< 70%) GFAP+ (20–30%) H3K27me3 Loss	*NF1*, *CDKN2A*/*CDKN2B*, and PRC2 core components (*EED* or *SUZ12*) mutations
**Dedifferentiated Liposarcoma** [67]	27–81 (mean age 52)	Equal	Retroperitoneum, spermatic cord and (more rarely) mediastinum, head and neck, and trunk.	Fascicles or sheets of atypical spindle cells along with lipogenic component with atypia	MDM2+, CDK4+, p16+, CD34+/−, STAT6−/+	MDM2 & CDK4 amplification
**Sarcomatoid Mesothelioma** [65]	41–94 (median 70)	22:1	Pleura, Peritoneum	Fascicles or haphazardly distributed atypical spindle cells with increase mitoses. Densely collagenized stroma with hypocellular atypical spindle cells in desmoplastic mesothelioma	CK+, EMA+, D2–40 +, Calretinin+, WT1 +	BAP mutation
**Soft Tissue Angiofibroma** [68]	Middle age, peak in 60th decade	0.75:1	Usually subcutis of extremities, particularly involving around large joints like knee	Variably myxoid to collagenous stroma, branching capillary network & uniform bland spindle cells with ovoid or tapering nuclei. Perivascular collagenization	EMA −/+, CD34 −/+	AHRR-NCOA2
**Spindle Cell Lipoma** [69]	40–60	10:1	Subcutis of posterior neck, upper back & shoulders Face, scalp, orbit, oral cavity & extremities rarely involved	Short fascicles of bland spindle cells with short stubby nuclei, variable number of adipocytes, and ropy collagen bundles. Fibromyxoid stroma, mast cells	CD34+, ASMA -, Desmin -, S100-	RB1 deletion
**Myofibroma** [70]	First decade (< 2 years) Adults	2:1 Equal	Skin & subcutis of extremities, head & neck & trunk. Infantile cases involve liver, heart, GIT, brain & bone	Distinctive biphasic pattern with nodules comprising of immature spindle cells in center with HPC-like vasculature and whorls of myoid cells at periphery with a basophilic pseudochondroid appearance	ASMA+/−, CD34 −/+, Desmin +	Nil
**Myofibroblastoma** [71]	50–60 (median 54)	2:1	Most cases involve Inguinal/groin area (vulva/vagina, perineum, and scrotum)	Short fascicles of spindle cells with short stubby nuclei, interspersed broad collagen bands & variable admixture of mature adipocytes	CD34+, Desmin + ASMA+/−	RB1 deletion
**Cellular Angiofibrom**a [71]	5th decade in women, 7th decade in men	Equal	Vulvovaginal/ inguinoscrotal & paratesticular region	Randomly distributed short, intersecting fascicles of spindle cells containing stubby nuclei. Scattered medium sized hyalinized vessels. Wispy stromal collagen	ER & PR + (50%), CD34+/−, Desmin −/+, ASMA −/+	RB1 deletion
**Deep Fibrous Histiocytoma** [21]	6–84 (median 37)	Slightly more in male	Extremities followed by head and neck region	Uniformly cellular storiform to short fascicular pattern of plump spindle cells. HPC-like branching vessels, stromal hyalinization	CD34 + (40%), ASMA f+/−, STAT6 -	Nil
**Dermatofibrosarcoma** **Protuberans [**75]	20–40	Slight male	Trunk, proximal extremities, head and neck region, genital area, the breast, and at acral sites	Dermal uniform spindle cells arranged in storiform, whorls & short fascicles. Infiltration of results in “honeycomb” appearance.	CD34+, ASAMA +/−, STAT6 -	COLIA-PDGFB
**Cellular Schwannoma** [66]	40–60	Equal	Paravertebral, retroperitoneum, pelvis & mediastinum	Predominantly or exclusively composed of Antoni A areas with interlacing fascicles of spindle cells having tapered nuclei. Hyalinized vessels are focally seen	S100+, SOX10+, STAT6-	NF2 mutations

Monophasic and poorly differentiated SSs mimic SFTs owing to sheet-like growth pattern, spindle to ovoid cell morphology, HPC-like vasculature in some cases and CD99 and BCL-2 expression [14]. Occasional cases may also show positivity for STAT6 [61, 62]. However, CD34 expression is almost always absent in SS. Some cases of SFT may also show weak expression of TLE1. However, strong and diffuse nuclear expression of TLE1 favors the diagnosis of SS. Molecular studies for translocation t(X;18) are recommended for confirmation of diagnosis [14, 66].

Malignant peripheral nerve sheath tumor (MPNST) is composed of cellular sheets of spindle cells which may alternate with hypocellular areas. Tumor cells may show HPC-like vasculature and accentuation around blood vessels. Some cases of low grade MPNST may also demonstrate positive expression for CD34. Majority of MPNSTs show typical “nerve sheath morphology” at least focally. Loss of H3K27me3, STAT6 negativity and expression of SOX-10, S100 or GFAP favors MPNST [21, 66].

Primary intrapulmonary SFTs are uncommon tumors which need to be differentiated from a number of mesenchymal, epithelial and mixed tumors. Intrapulmonary SFTs especially need to be distinguished from sarcomatoid carcinoma. Squamous cell carcinoma, carcinoid tumor and malignant mesothelioma (originating from pleura) may also show spindle cell morphology and mimic SFT [24]. Multifocal expression for cytokeratins, observed in some pleural and abdominal SFTs, can be misleading as two important differential diagnoses in these locations (sarcomatoid carcinoma and mesothelioma) also show similar staining pattern [65]. Other mesenchymal intrapulmonary tumors such as inflammatory myofibroblastic tumor (IMT), leiomyosarcoma, leiomyoma, and adenofibroma should also be considered in the differential diagnosis (DD). Positive expression for CD34 and STAT6 IHC stains helps in establishing the diagnosis of SFT [24, 61, 62].

Fat-forming or lipomatous SFT can resemble benign lipomatous tumors such as spindle cell lipoma (SCL) or malignant lipomatous tumors such as WDL or DDL. Spindle cell lipoma exhibits bland spindle cells, ropy collagen bundles and mature adipose tissue and expresses CD34 immunostain. All these features are seen to a variable degree in lipomatous SFT. However, STAT6 expression is not observed in SCL. RB1 gene loss by fluorescence in situ hybridization (FISH) is also observed in SCL.

DDLs exhibit a wide variety of histological features which resemble low- and high-grade sarcomas. In retroperitoneal location especially, DDL can resemble malignant fat-forming SFT. The situation is further complicated by STAT6 nuclear expression in DDL. Mouse double minute 2 (MDM2) gene amplification and diffuse nuclear IHC expression in DDL is extremely helpful in this situation because of 100% specificity and 97.2% sensitivity. CDK4 and p16 are also useful IHC markers for the diagnosis of WDL and DDL [31–34, 61, 66, 67, 69, 71–73].

Cellular schwannomas can also resemble SFT as these are composed of fascicles of bland spindle shape cells against a collagenous background and frequently exhibit thick-walled and hyalinized blood vessels. Some cases may show CD34 expression. Presence of thick fibrous capsule, foamy macrophages, lymphoid infiltrate, wavy nuclei with tapered ends and S100 expression favors the diagnosis of schwannoma [66].

Angiofibroma of soft tissue is a circumscribed tumor composed of bland spindle cells against variably collagenous to myxoid background stroma containing a rich network of thin-walled blood vessels. Tumor cells are variably positive for CD34, EMA and Desmin. STAT6 expression is typically negative. The molecular signature of this tumor is translocation t(5;8) (p15;q13) resulting in AHRR-NCOA2 fusion gene [68].

Myofibromas are biphasic tumors occurring in young age which can resemble SFT due to pericytic growth pattern and presence of HPC-like vasculature. These tumors variably express CD34 and ASMA. Presence of myoid nodules at the periphery of the tumor is unique to myofibroma and provides a useful diagnostic clue [70].

Myofibroblastomas are circumscribed tumors composed of intersecting fascicles of bland spindle shaped myofibroblastic cells with intervening collagen bundles and occasional mature adipocytes. Tumor cells demonstrate positivity for CD34, Desmin and ASMA IHC stains. These tumors also show RB1 gene loss by FISH which is not observed in SFT [71].

Cellular angiofibroma is another well circumscribed, multilobulated tumor composed of spindle cells against a collagenous and edematous background stroma containing small to medium sized thick walled and hyalinized blood vessels. Some cases also show mature adipocytes and collagen bundles. Variable expression for CD34, ASMA and Desmin may be seen. RB1 gene loss is also observed [71].

Deep fibrous histiocytoma also exhibits intersecting fascicles or storiform pattern of bland spindle cells. Stromal hyalinization and HPC-like vessels are also seen. Tumor cells may show positive expression for CD34 and ASMA but STAT6 is negative [21].

In superficial locations, two soft tissue tumors with CD34 expression need to be distinguished from SFT [22]. These tumors are DFSP and superficial CD34 positive fibroblastic tumor (SCD34PFD). DFSP is a dermal based tumor usually composed of fairly uniform spindle cells with elongated nuclei arranged in storiform pattern and infiltrating into the subcutaneous adipose tissue. SCD34PFD is also a dermal based tumor comprising of sheets and fascicles of spindle to epithelioid cells. Tumor cells have moderate cytoplasm and may show moderate to marked atypia [74]. In comparison to these tumors, SFTs have relatively more defined borders and heterogeneous cellularity. Molecular studies on DFSP typically show translocation t(17;22) and/or supernumerary ring chromosome r(17;22) [75].

In abdominal (and less commonly extra-abdominal) locations, one important lesion which should be considered in the DD is low grade gastrointestinal stromal tumor (GIST) which often shows spindle to ovoid cells arranged in fascicles or randomly distributed. In addition, CD34 expression is shared by GISTs and SFTs. However, majority of GISTs are positive for CD117 and DOG1 while STAT6 is always negative [14].

In meninges, meningothelial meningioma can be easily distinguished from SFT by epithelioid cell morphology, whorling, syncytial pattern and psammomatous calcifications [76]. Fibrous meningioma can resemble SFT due to spindle cell morphology and vague fascicular pattern. Atypical and anaplastic meningioma can mimic malignant SFT due to presence of patternless/sheet-like growth, necrosis, frequent mitoses, nucleolar prominence, nuclear pleomorphism and small cell change. Meningiomas usually express positivity for EMA and progesterone receptor (PR). This expression is either completely or partially lost in atypical and anaplastic cases. However, all types of meningioma are negative for CD34 and STAT6 IHC stains [76]. Therefore, a panel of these IHC stains should be applied in meningeal tumors when SFT is considered in the DD [77].

In hypercellular SFTs, tumor cells usually acquire round cell morphology and mimic small round cell sarcomas such as Ewing sarcoma (ES), rhabdomyosarcoma and mesenchymal chondrosarcoma (MC) [25]. Due to high incidence of rhabdomyosarcoma in young patients with head and neck tumors, it should be kept in the differential diagnosis. Presence of cambium layer and rhabdomyoblastic differentiation along with absence of HPC-like vasculature are useful clues in favor of rhabdomyosarcoma. In addition, myogenic markers like MyoD1, myogenin and desmin are highly sensitive and specific markers for rhabdomyosarcoma and are usually not expressed in SFT [21, 78, 79].

The round cell population of mesenchymal chondrosarcoma also shows background HPC-like vasculature which may be indistinguishable from SFT on morphology. This raises a diagnostic challenge if chondroid component is not sampled in small biopsy specimens. Both ES and MC like SFT express CD99. Positivity for NKX2.2 which is a recently introduced sensitive and specific IHC marker for ES and expression of CD34 and STAT6 by SFTs help in reaching the correct diagnosis [21, 25].

Myxoid change in SFT is well reported and a number of other soft tissue tumors with myxoid features must be excluded. These tumors include low grade myxofibrosarcoma, low grade fibromyxoid sarcoma and myxoid liposarcoma [25–27, 55, 60, 80, 81]. Some of these tumors may express CD34 and occasional cases with STAT6 expression have also been reported. Careful examination of tumor for cases showing conventional SFT morphology along with diffuse and strong nuclear STAT6 expression leads to accurate diagnosis [61, 62].

Soft tissue tumors with epithelioid morphology such as epithelioid sarcoma and epithelioid angiosarcoma sometimes need to be considered in the DD [25]. SFT with giant cells is a rare occurrence and the DD of this morphological form include soft tissue sarcomas with giant cell component and nodular fasciitis [25, 82].

Prostatic stromal tumors of uncertain malignant potential (STUMP) and prostatic stromal sarcoma (PSS) can pose diagnostic problems for pathologists. STUMP exhibits haphazard fascicles of spindle cells which do not exhibit significant nuclear atypia or mitoses. However, PSS exhibits solid growth of spindle to epithelioid cells with nuclear atypia, increased mitoses and necrosis. However, these demonstrate a more regular pattern histologically, lack HPC-like vasculature and collagen deposition [55, 83]. Some of these tumors may express positivity for CD34 and some of the prostatic SFTs may also express PR [83]. However, STUMP and PSS lack nuclear expression for STAT6. Combined specificity of STAT6 and ALDH1 is 100% for SFTs of this region [55].

### Molecular alterations in SFT

A number of studies have identified recurrent fusion of two genes in majority of SFTs by next generation sequencing (NGS) and RT-PCR techniques. This gene fusion is considered to be an initial event in the tumorigenesis of SFT [12, 20]. These two genes, NGFI-A binding protein 2 (NAB2) and STAT6 are located very close to each other on chromosome 12 [10–12]. Therefore, their fusion is difficult to detect by FISH technique [12]. In a study conducted by Barthelme et al., diffuse and strong nuclear IHC expression for STAT6 was observed in 100% cases while gene fusion was detected in 92% cases by RT-PCR. Lack of gene fusion detection was not related to technical issues in RNA isolation from formalin fixed tissue [20].

NAB2 is a transcriptional repressor while STAT6 is a transcription factor. Both proteins play important roles in regulation of inflammation, fibroblastic activation, collagen formation and vessel formation [84, 85]. These proteins affect early growth response 1 (EGR1) transcription factor which is an important regulator of fibrosis and wound healing in opposing manner [85–87]. NAB2 gene possesses 7 exons while STAT6 gene possesses 23 exons. As a result, a number of fusion variants are generated with variable frequencies [10–12].

In a study of 52 pleural and extra-pleural tumors, 12 fusion variants were identified by multiplex RT-PCR in 48 (92%) cases. Three fusion variants were more frequent and accounted for 75% of all fusion variants. Fusion variants were grouped according to their potential functional effects among the predicted chimeric proteins. These fusion categories significantly correlated with patient age, tumor size, mitotic activity, anatomic site, histomorphological classification and clinical follow up. Majority of the tumors harboring NAB2ex4-STAT6ex2/3 gene fusion variant involved thoracic cavity and showed classic fibrous SFT morphology. When compared with tumors having other fusion variants, these tumors had higher tumor age, larger tumor size, lower mitotic activity and lower recurrence rate. Majority of the tumor harboring NAB2ex6-STAT6ex16 and NAB2ex6-STAT6ex17 fusion variants were deep seated and involved extra-thoracic sites. These tumors occurred in significantly younger age group, were smaller in size, showed higher mitotic activity, cellular SFT or typical HPC type morphology and higher recurrence rate [20].

Robinson et al. also found that majority of the tumors with gene fusion variant NAB2ex6-STAT6ex16/17 were extra-thoracic in location while two out of three tumors with NAB2ex4-STAT6ex2/3 gene fusion variant were seen in pleuropulmonary location [10]. In a study by Chmielecki et al., majority of the tumors with NAB2ex4-STAT6ex2 gene fusion variant involved lung or pleura. NAB2ex6-STAT6ex16/17 gene fusion variant was not detected [11]. Mohajeri et al. observed that the majority of tumors with NAB2ex6-STAT6ex16/17 gene fusion variants were located in extra-thoracic regions while half of the tumors with NAB2ex4-STAT6ex2 gene fusion variant involved pleuropulmonary region [12]. Yamada et al. and Park et al. did not find any direct association of gene fusion variants with malignancy. However, they observed that the association between gene fusion variants and tumor location could indirectly affect the biological behavior of SFT [43, 50].

TERT promoter mutations were found to be associated with malignant SFTs in two studies [50, 51]. A comprehensive genetic analysis in that study also revealed down regulation of target genes of EGR1 [12]. Overexpression of genes encoding stem cell markers such as ALDH1 has also been demonstrated. ALDH1 has been related to poor prognosis in breast carcinoma. In addition, a number of growth factors and kinases are overexpressed in SFTs. These include PDGFα, PDGFβ, PDGFR-α, PDGFR-β, IGF1R, EGFR, VEGF, IGF2, c-Met, c-kit, c-erbB2, PTEN, phosphorylated (p) AKT, pS6, p4EBPEGFR, ERBB2, FGFR1, and JAK2 [88]. Overexpression of these markers leads to activation of Akt/mTOR pathway and appears to be associated with tumor necrosis [89]. Targeting Akt/mTOR pathway and IGF signaling pathway might prove beneficial for irresectable tumors [89]. PDGFR-α is more frequently expressed in malignant SFTs compared to more localized tumors [90]. A number of studies have also reported complete loss or partial deletion of chromosomes 1,6,9,13,15,17,18, X and gain of chromosomes 5,8,13,21 [91–94]. In a recent study, TP53 mutations were identified in 41% malignant SFTs [50]. Dedifferentiated SFTs also characteristically show TP53 mutations [25, 41–43, 95].

### Follow up and prognosis

In one study, malignant SFTs were related with higher rate of local recurrence and distant metastasis [96]. In another study, tumors with malignant histological features demonstrated indolent behavior while tumors lacking malignant histological features behaved aggressively [44, 47]. Extrathoracic site is an independent predictor of poor prognosis. Tumors located in meninges, pelvis, retroperitoneum and mediastinum are associated with greater risk of recurrence [46]. Despite surgical excision with clear margins, local recurrence and distant metastasis can occur in some cases [13]. In one study, local recurrence was observed in 13 (39.3%) out of 39 cases [20]. Distant metastasis is a predictor of poor prognosis and 75% patients with metastasis die of their disease [97]. In a study of extra-thoracic SFTs, median overall survival duration ranged from 59 to 94 months and 5-year and 10-year survival rates were 89 and 73% respectively [49].

### Treatment

Wide surgical excision is the mainstay of treatment and adjuvant radiotherapy and chemotherapy are not required in routine cases [98]. However, adjuvant radiotherapy has been suggested to improve the local control of tumor [99]. In meningeal tumors, SFTs (WHO grade 1) are treated with surgery alone while HPCs (WHO grades 2 & 3) benefit from adjuvant radiotherapy [15]. Due to their rarity and lack of randomized control trials, there is no global consensus on treatment of SFTs. A multidisciplinary team approach is recommended for treatment and management of these tumors [19].

## Conclusions

Accurate diagnosis is essential for appropriate treatment and management of SFTs. NAB2-STAT6 gene fusion and its IHC expression are consistently observed in these tumors. Immunohistochemistry is the most sensitive and specific means of diagnosing SFT and is practical and economical as well. Molecular studies require expensive equipment and well-trained staff which reduces their practicality and feasibility in resource limited laboratories of developing countries. Molecular testing may be helpful where IHC results are ambiguous. Thorough knowledge about the morphological variations of SFTs and correlation with clinical, IHC and molecular features are helpful in avoiding misdiagnosis.

## Data Availability

Data and materials of this work are available from the corresponding author on reasonable request.

## Abbreviations

SFT: Solitary fibrous
MPNST: Malignant peripheral nerve sheath tumor
IHC: Immunohistochemical
HPC: Hemangiopericytoma
WHO: World health Organization
SS: Synovial sarcoma
STUMP: Smooth muscle tumors of uncertain malignant potential
DFSP: Dermatofibrosarcoma protuberans
EMA: Epithelial membrane antigen
ASMA: Alpha smooth muscle actin
GFAP: Glial fibrillary acidic protein
NSE: Neuron-specific enolase
IMT: Myofibroblastic tumor
DD: Differential diagnoses
SCL: Spindle cell lipoma
WDL: Well differentiated liposarcoma
DDL: Dedifferentiated liposarcomas
GIST: Gastrointestinal stromal tumor
MC: Mesenchymal chondrosarcoma
PSS: Prostatic stromal sarcoma
NGS: Next generation sequencing
RT-PCR: Reverse transcription polymerase chain reaction
NAB2: NGFI-A binding protein 2
STAT6: Signal transducer and activator of transcription 6
FISH: Fluorescence in situ hybridization
ALDH1: Aldehyde dehydrogenase 1
